# Fibroblasts in intestinal homeostasis, damage, and repair

**DOI:** 10.3389/fimmu.2022.924866

**Published:** 2022-08-10

**Authors:** Niki Chalkidi, Christina Paraskeva, Vasiliki Koliaraki

**Affiliations:** Institute for Fundamental Biomedical Research, Biomedical Sciences Research Center “Alexander Fleming”, Vari, Greece

**Keywords:** mesenchymal cells, heterogeneity, epithelial homeostasis, tissue injury, regeneration, immune responses

## Abstract

The mammalian intestine is a self-renewing tissue that ensures nutrient absorption while acting as a barrier against environmental insults. This is achieved by mature intestinal epithelial cells, the renewing capacity of intestinal stem cells at the base of the crypts, the development of immune tolerance, and the regulatory functions of stromal cells. Upon intestinal injury or inflammation, this tightly regulated mucosal homeostasis is disrupted and is followed by a series of events that lead to tissue repair and the restoration of organ function. It is now well established that fibroblasts play significant roles both in the maintenance of epithelial and immune homeostasis in the intestine and the response to tissue damage mainly through the secretion of a variety of soluble mediators and ligands and the remodeling of the extracellular matrix. In addition, recent advances in single-cell transcriptomics have revealed an unexpected heterogeneity of fibroblasts that comprise distinct cell subsets in normal and inflammatory conditions, indicative of diverse functions. However, there is still little consensus on the number, terminology, and functional properties of these subsets. Moreover, it is still unclear how individual fibroblast subsets can regulate intestinal repair processes and what is their impact on the pathogenesis of inflammatory bowel disease. In this mini-review, we aim to provide a concise overview of recent advances in the field, that we believe will help clarify current concepts on fibroblast heterogeneity and functions and advance our understanding of the contribution of fibroblasts in intestinal damage and repair.

## 1 Introduction

The mammalian intestine is responsible for nutrient and water absorption, but is also constantly exposed to environmental factors, including commensal and pathogenic microbes, food products and toxins. It has thus developed intricate cellular and molecular mechanisms to ensure tissue homeostasis and normal organ function. Among them is the organization in villi and crypts, which are lined by a single epithelial layer that self-renews every 5-7 days. This is mediated by intestinal stem cells (ISCs) at the bottom of the crypts, which differentiate into functionally distinct epithelial populations that move towards the top, where they will be eventually shed. This process is maintained by both intrinsic and extrinsic mechanisms, including paracrine signals from neighboring stromal cells (1). Besides epithelial homeostasis, the development of immune tolerance and a symbiotic relationship with the microbiota is of paramount importance for host health and is mediated by resident immune cells and specialized lymphoid structures (2). Finally, a broad blood and lymphatic vessel network ensures blood and oxygen transfer to the intestine, as well as transport of nutrients and the removal of interstitial fluid (3, 4).

During the last decade, the role of mesenchymal cells in the maintenance of intestinal homeostasis has gained momentum (5). Smooth muscle cells constitute the muscularis, and lamina propria fibroblasts produce and remodel the extracellular matrix (ECM) to support intestinal structure and integrity. Fibroblasts also play significant roles in epithelial stem cell maintenance and differentiation, immune homeostasis, and endothelial cell functions (6, 7). Recent data have revealed novel fibroblast-specific mechanisms and an unanticipated heterogeneity, which is dependent on the distinct expression profile and location of each subset (5). In this mini review, we will provide a concise overview of recent findings on fibroblast heterogeneity and functions in intestinal homeostasis, damage, and regeneration.

## 2 Main text

### 2.1 Fibroblast heterogeneity and functions in intestinal homeostasis

**Figure 1:**
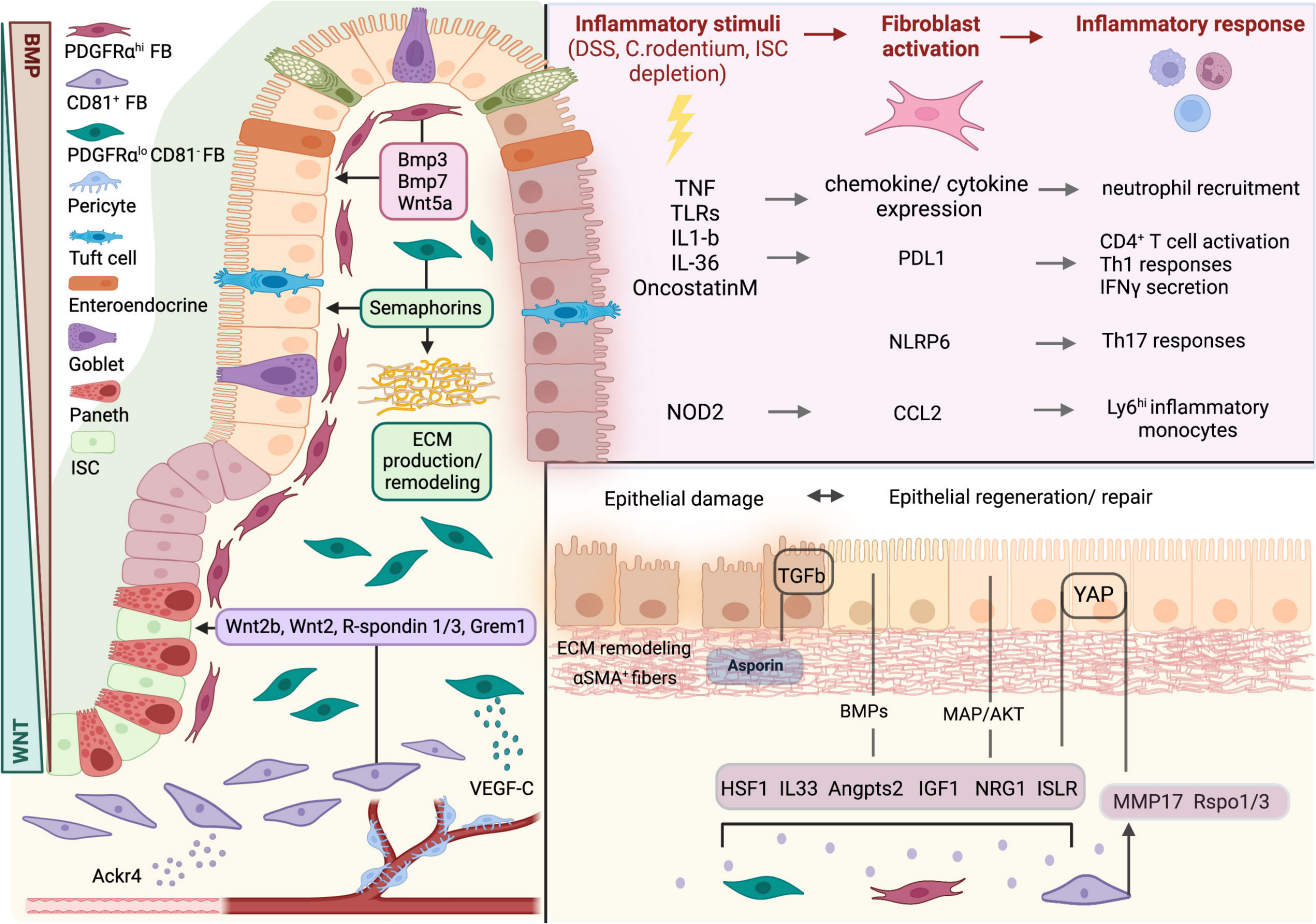
Fibroblasts in intestinal homeostasis, damage, and repair. Intestinal homeostasis is regulated by 3 distinct fibroblast subsets through the production of effector molecules. WNT ligands, R-spondins and Gremlin 1 are produced by CD81^+^ fibroblasts and maintain intestinal stem cell (ISC) identity. PDGFRα^hi^ fibroblasts orchestrate epithelial differentiation through the production of BMPs and WNT5A. In the lamina propria, PDGFRα^lo^CD81^-^ fibroblasts contribute to extracellular matrix (ECM) production and remodeling. Upon inflammatory stimuli, fibroblasts are activated and secrete a variety of pro-inflammatory factors to drive immune cell recruitment and function. During damage, intestinal fibroblasts provide paracrine signals to promote epithelial regeneration and ECM remodeling. FB, fibroblast; ISC, intestinal stem cell. Created with BioRender.com.

Recent advances in single cell transcriptomics, lineage tracing approaches, and genetic targeting have revealed the extent and functional significance of intestinal fibroblast heterogeneity. However, there is still little consensus on the number of fibroblast subpopulations, their terminology, and functions. Comparative analysis of single cell RNA sequencing studies of the mouse intestine based on marker gene expression points to the presence of three main functionally distinct fibroblast subsets, similar to the ones described by McCarthy et al., which re-analyzed results from four such studies on a common computational platform (5) (**Table 1**; **Figure 1**). These subsets include:

**Table 1:** Comparison of fibroblast subsets from recent single cell RNA sequencing analyses.

Intestinal region/pathology	Analyzed population	Populations/Subsets										Ref
*Mouse*												
SI - Healthy	PDGFR1^+^	PDGFRα^hi^ telocytes		Lo-1 FB	Lo-2 FB							(8)
SI - Healthy	PDGFRB^+^	FB5		FB4	FB3/FB1	FB2/FB1				SMCs	Mural cells	(9)
SI - Healthy	EpCAM^-^CD45^-^Ter119^-^CD31^-^BP3^-^	PDGFRα^hi^		PDGFRα^lo^CD34^hi^CD81^+^	PDGFRα^lo^ CD34^lo^ Igfbp5^+^	PDGFRα^lo^ CD34^lo^ Fgfr2^+^				SMCs	Pericytes	(10)
Colon - Healthy	EpCAM^-^CD45^-^	CTFs		CBF2	CBF1			MFs/SMCs			Pericytes	(11)
Colon - Healthy	EpCAM^-^CD45^-^	F3/F4		F1	F2			F4		SMCs	Pericytes 1/2	(12)
Colon - Healthy	EpCAM^-^CD45^-^Ter119^-^	FB2 - MFs		FB3 - Interstitial	FB1 - MAFs			FB2 - MFs				(13)
Colon - Healthy	EpCAM^-^CD45^-^Ter119^-^CD31^-^BP3^-^	PDGFRα^hi^		PDGFRα^lo^CD34^+^CD81^+^	PDGFRα^lo^ CD34^+^ CD90^+^	PDGFRα^lo^ CD34^+^ Fgfr2^+^		MFs		SMCs	Pericytes	(10)
Colon/SI - Healthy	Datasets from (10, 14)	PDGFRα^hi^		*pi16* ^+^	*Col15a1* ^+^	*Fbln1* ^+^						(15)
Whole intestine	Bapx1^+^ stromal cells	*Ednrb1* ^hi^		*Ackr4* ^hi^/*Has1* ^hi^	*C1qtnt3* ^hi^/*Dkk2* ^hi^	*Cxcl5* ^+^/*Dkk2* ^hi^		*Cxcl5* ^+^/Pericyte like			*Rgs4* ^hi^ Pericytes	(16)
DSS – acute (D3)	CD90^med^	*Foxl1* ^+^ telocytes		C5 - MRISCs				MFs				(17)
Colon – Healthy/ DSS – acute (D7)	EpCAM^-^CD45^-^	S2		S3	S1		S4 – IAFs	MFs			Pericytes	(14)
Colon – Healthy/DSS/acute (D8)	EpCAM^-^CD45^-^	CTFs		Matrix FB 2	Matrix FB 1			MFs/SMCs			Pericytes	(18)
DSS – acute (D7)	Dataset from (14)			*pi16* ^+^	*Col15a1* ^+^		*Adamdec1* ^+^ *Lrcc15* ^+^					(15)
DSS - chronic	EpCAM^-^CD45^-^Ter119^-^	FB2 - MFs		FB3 - Interstitial	FB1 - MAFs			FB2 - MFs				(13)
***Human***												
Colon – Healthy/colitis	EpCAM^-^ CD45^-^ CD235^-^	S2a	S2b	S3	S1		S4 – IAFs	MFs/SMCs			Pericytes	(14)
Colon – Healthy/colitis	Lamina propria non epithelial	*WNT5B* ^+^_1	*WNT5B* ^+^_2	*WNT2B* ^+^ *RSPO3* ^+^	*WNT2B* ^+^ *Fos* ^hi^	*WNT2B* ^+^ *Fos* ^lo^	IAFs	MFs/SMCs			Pericytes	(19)
Inflamed/non-inflamed tissue	Dissociated tissue	PDGFRα^+^ FB		ABCA8^+^ fibroblasts			IAFs	MFs/SMCs			Pericytes	(20)
Colon (pediatric) – Healthy/IBD (UC/CD)	CD45^-^	FB epithelia proxima		FB TACI/WNT2B^hi^	FB LP/TACI		IAFs	MFs			Pericytes	(21)
Colon - Normal	Dissociated tissue	S2		S3	S1			MFs	SMCs		Pericytes	(22)
Colon - Normal	EDTA-treated tissue	*ICAM1* ^+/-^ telocytes		*CD24* ^+^/*NT5E* ^+^ FBs	*FGFR2* ^+^ FBs			*DES* ^+^/*MFAP* ^+^ MFs			Pericytes	(23)

CTF, crypt-top fibroblast; CBF, crypt-bottom fibroblast; FB, fibroblasts; IAF, inflammatory fibroblast; IBD, inflammatory bowel disease; LP, lamina propria; MAF, mucosal-associated fibroblast; MF, myofibroblast; S, stromal; SI, small intestine; SMC, smooth muscle cell.

**CD81^+^ fibroblasts** (10), also called trophocytes (5), crypt-bottom fibroblasts (CBFs) (11), MAP3K2-regulated intestinal stromal cells (MRISCs) (17) or *pi16*+ fibroblasts (15). They are located within the submucosa, near vascular structures and below crypts, and are the primary cellular source of WNTs (e.g. *Wnt2* and *Wnt2b*), the BMP antagonist Gremlin 1, and R-spondins (8–10, 15, 17). They mainly function to maintain intestinal stem cell identity and proliferation. *In vitro*, CD81^+^ trophocytes provide support for intestinal organoid expansion and *in vivo* ablation of *Grem1* ^+^ cells results in extensive intestinal stem cell loss (8). In addition, they express the atypical chemokine receptor Ackr4, which marks a distinct fibroblast population that regulates endothelial cells functions (24).

**PDGFRα^hi^ fibroblasts ** (10, 15), also called telocytes (8, 17), crypt-top fibroblasts (CTFs) (11, 18) and *Ednrb* ^hi^ fibroblasts (16). They are characterized by expression of high levels of *PDGFRα*, BMPs, among which *Bmp3* and *Bmp7* are uniquely expressed, *Wnt5a*, *F3*, *Sox6, Foxl1*, and low levels of *Acta2* (8–12, 14–18). They are localized directly under the epithelial layer and are concentrated at the top of crypts and villi (8, 10, 11, 13, 15, 25). They may also include subepithelial myofibroblasts, as they express αSMA (5, 13). The expression of BMPs and their location suggests an important function in epithelial cell differentiation (26–28). Therefore, the relative location of CD81^+^ and PDGFRα^hi^ fibroblast subsets contributes to the generation of a signaling gradient along the small intestinal villous-crypt and colonic crypt top-bottom axis that facilitates ISC maintenance and differentiation (5). Studies using constitutive and conditional *Foxl1*-Cre strains and immunoelectron microscopy have shown that *Foxl1* ^+^ cells form a subepithelial plexus along the entire villous/crypt axis and exhibit unique structural characteristics, including long processes called ‘telopodes’, thus leading to the term ‘telocytes’. However, *Foxl1* ^+^ cells also express stem cell trophic factors, such as *Wnt2b* and *Rspo3*, as well as *Sfrp1* and *Grem1*, which are markers of CD81^+^ fibroblasts (29, 30). *Foxl1* ^+^ cell depletion or cell-specific deletion of WNT secretion leads to marked changes in the epithelial architecture, including reduced villi length and crypt depth, and a reduction in stem and progenitor cell proliferation (29, 30). These results indicate that telocytes targeted by the *Foxl1*-Cre mice could include both PDGFRα^hi^ and pericryptal fibroblasts to some extent. Indeed, a recent study differentiated between crypt and *Lgr5* ^+^ villous tip telocytes (VTTs), and ablation of the latter led to changes in epithelial gene expression at the villus tip, but did not have the detrimental effects of *Foxl1* ^+^ cell depletion (31). We also recently showed that Col6a1-Cre mice target the entirety of PDGFRα^hi^ fibroblasts, along with pericytes and a small number of PDGFRα^lo^ cells. Depletion of this population in the middle/distal colon did not disrupt intestinal morphology, but led to altered distribution of proliferating epithelial cell and reduced enteroendocrine numbers (25). The differences between these experiments most probably reflect the exact specificities of each strain and should be carefully considered.

**PDGFRα^lo^CD81^-^ fibroblasts**, which reside in the lamina propria, around crypts and inside the villous core (9, 10). They can be further divided into at least two subsets that express Col15a1, Igfbp5/CD90 (small intestine/colon) and Fgfr2, Fbln, respectively (9, 10, 15). They secrete basement membrane proteins and contribute to ECM production and remodeling (15). They also maintain lacteal integrity and function through YAP/TAZ-mediated VEGF-C secretion (9). Notably, CD90^+^ cells have been shown to support epithelial cell growth through the production of class 3 semaphorins (32).

Additional mesenchymal subsets include *Pdgfra^-^NG2^+^Rgs5* ^+^ pericytes surrounding blood vessels and capillaries (33), smooth muscle cells (SMCs) around blood vessels and lymphatic lacteals and in the muscle layer, and myofibroblasts. Varying levels of *Acta2*, *Myh11* and *Des* can help with the distinction between SMCs and myofibroblasts, but the two terms are sometimes used interchangeably in single cell RNA sequencing analyses (9–12, 14, 16, 18). Notably, the small intestine and colon display similar mesenchymal subsets with location-specific differences in their transcriptional profiles (5, 10).

Besides the regulation of epithelial homeostasis and tissue integrity, pseudotime analysis and lineage inference have indicated that CD81^+^/*pi16* ^+^ fibroblasts could also act as mesenchymal stem cells and thus as sources of adult fibroblasts, which pass through intermediate PDGFRα^lo^CD81^-^Col15a1^+^/CD90^+^ cells towards differentiated subsets (10, 14, 15). This is in accordance with lineage tracing data of *Grem1* ^+^ cells, which can renew the entire mesenchymal sheath over a year (34). We also recently showed that following depletion of Col6a1-Cre^+^ colonic fibroblasts, CD34^+^ cells could proliferate, occupy subepithelial locations and alter their gene expression profile to support epithelial cell differentiation and regeneration, highlighting the potential plasticity of resident fibroblasts (25).

There is fewer insight into the significance of distinct fibroblast subsets in the regulation of intestinal immune homeostasis. Of note, there are also specialized stromal populations that regulate immunity within the topologically restricted structures of the gut-associated lymphoid tissue, including Peyer’s patches and isolated lymphoid follicles (6). Still, intestinal fibroblasts, and especially PDGFRα^hi^ and PDGFRα^lo^CD81^-^ cells, express various chemoattractants, cytokines and cytokine receptors and could thus regulate immune cell turnover and function (10, 35, 36). Fibroblasts also produce retinoic acid, which synergistically with GM-CSF drives the functional education of migratory dendritic cells (37). BAFF production by lamina propria fibroblasts induces B cell proliferation and differentiation to IgA^+^ plasma cells (38). Human colonic fibroblasts express PD-L1 and PD-L2, which suppress CD4^+^ T-helper cell activation and proliferation through inhibition of IL-2 production (39). Conversely, they also express MHC-II molecules and CD80/86 co-stimulators, suggesting a potential role as non-professional antigen presenting cells, which can stimulate allogeneic CD4^+^ T-cell proliferation (40) and induce activation of Tregs at least *in vitro* (41).

Distinct fibroblast subsets have also been found in the human intestine, and share many similarities with their mouse counterparts, as shown both by direct comparison of single cell transcriptomic data (11, 14, 18) and assessment of marker gene expression (**Table 1**). Among them *PDGFRA* ^+^, *WNT5B* ^+^, S2, or epithelia proxima fibroblasts express *FOXL1*, *WNT5A*, and BMPs, and display a subepithelial localization, correlating with mouse PDGFRα^hi^ fibroblasts (14, 19–23). Interestingly, in humans, two clusters have been identified, one expressing *ACTA2* and *TAGLN* and the other *PTX3*, *NPY*, but their potential distinct functions are yet unknown (14, 19). Additionally, *WNT2B* ^+^ cells that express *RSPO3* are most likely equivalent to mouse CD81^+^ fibroblasts, while *WNT2B* ^+^ *FOS* ^+^ lamina propria fibroblasts correlate with PDGFRα^lo^CD81^-^ fibroblasts (14, 19, 21). Additional subsets include myofibroblasts/smooth muscle cells and pericytes (14, 19–23). These results further support the value and translatability of mouse studies in modeling human health and disease in the gut.

### 2.2 Fibroblasts in intestinal damage and repair

Tissue damage or infection leads to inflammation so that damaged cells and microbes can be removed and is followed by the resolution of inflammation and epithelial regeneration to restore organ function. Deregulation of the mechanisms underlying these processes can lead to pathology, including chronic inflammation, fibrosis, and cancer. Recent data show that resident intestinal fibroblasts play a significant role both in the support of initial immune responses and in the resolution of inflammation, the remodeling of the ECM and the re-organization of the intestinal epithelium, including the re-epithelization of the tissue in ulceration sites (35) (**Figure 1**).

#### 2.2.1 Insights from single-cell transcriptomics

All single cell transcriptomic data related to intestinal damage, inflammation and repair in the mouse to date originate from analyses of the DSS colitis model (42). A recent such analysis of the colon at different timepoints during the acute damage and repair phases showed that fibroblasts have the highest impact on other cells and could act as a hub of cellular interactions during acute inflammation (43). Fibroblast-specific studies, which include early non-inflamed (day 3), acute severely inflamed (day 7-8) and chronically inflamed timepoints reveal the persistence of homeostatic subsets, which maintain their topology, although their gene expression is altered (13, 14, 17, 18, 25, 44). For example, production of BMPs by PDGFRα^hi^ fibroblasts is reduced, while *Grem1*, *Rspo3* and *Sfrp1* are induced in all PDGFRα^lo^ cells during acute colitis, indicating a shift towards the support of ISC proliferation (14, 18). The most prominent changes though involve the significant increase in inflammatory mediators, ECM components and remodeling enzymes (13, 14, 18). One study further reported the identification of a distinct inflammatory fibroblast subset that showed increased expression of cytokines and chemokines, such as *Il33* and *Ccl19* (14). However, this could also represent an activated state of CD81^+^ fibroblasts, as indicated by the proximity and combined analysis of the two subsets. Additional subset-specific changes include the overexpression of *Il33*, *Il6* and *Ptx3* in CD81^+^ cells, *Grem1*, *Il11* and *Mmp3* in PDGFRα^lo^ cells, and *Cxcl13* and *Timp3* in PDGFRα^hi^ fibroblasts, but their potential context- and/or location-specific basis and their functional significance is not clear (14). Re-analysis of the same data in the context of a pan-tissue inflammatory framework revealed the persistence of the same pan-tissue homeostatic fibroblasts, and further identified the emergence of an *Lrcc15* ^+^ myofibroblast-specific subset and a “colitis-specific” *Adamdec1* ^+^ subset with increased *Grem1* and *Il11* expression, which also included BMPs, *Mmp3* and *Timp3* (15). In both studies, CD81^+^/*pi16* ^+^ showed increased proliferation/stemness, indicative of their potential as cellular sources of activated fibroblasts, while PDGFRα^hi^ cells were proportionally reduced (14, 15). In chronic inflammation, immune-related genes expressed in fibroblasts also include those encoding complement components, MHC-related molecules, redox regulators, and chemokines (13). Notably, CD81^+^ cells show increased frequency and higher expression of pro-inflammatory genes, including the pro-fibrotic cytokine *Il11*, in line with the acute colitis data (13).

Similar analyses in patients with ulcerative colitis and Crohn’s disease identified both homeostatic subpopulations and an additional inflammation-associated subset (IAFs) that was enriched in pro-inflammatory genes, including cytokines (e.g. *IL11*, *IL33*, *IL6*), and chemokines (e.g. *CCL19*, *CXCL1/2/3/4/5/8*). The presence of *WNT2B* ^+^ and *WNT5B* ^+^ subsets in IAFs (19), and the expression of inflammatory genes in homeostatic subsets (20, 21) support the hypothesis that IAFs represent an activated state of diverse fibroblast subpopulations. Together, these findings suggest similarities in fibroblasts remodeling during damage and inflammation in humans and mice.

#### 2.2.2 Fibroblasts in epithelial regeneration

Single cell transcriptomic data and *in vitro*/*in vivo* experiments show that fibroblasts play significant roles in the regulation of epithelial responses during tissue damage and repair. Both broad and subset-specific mechanisms related to the expression profile and location of fibroblasts have been identified. CD81^+^ fibroblasts contribute to intestinal repair through their increased production of Wnts and R-spondins 1 and 3 upon damage (44, 45). Fibroblast-derived R-spondin 3, in particular, is required for tissue repair after DSS-induced damage, DT-induced ISC ablation or *C.rodentium* infection (46–48). It is activated by IL-1R1 signaling and regulates stem cell renewal, barrier restoration and de-differentiation of Axin2^-^ cells, depending on the mouse model. R-spondin 1 is also increased during DSS-colitis, through a mechanism involving reactive oxygen spieces (ROS)-mediated activation of an MAP3K2/ERK5/KLF2 axis, and acts to protect the stem cell pool (17). Recently, membrane-bound MMP17, expressed by *Grem1* ^+^ mesenchymal cells, was also shown to be required for epithelial restoration following DSS- or irradiation-induced damage though cleavage of periostin and activation of YAP in epithelial cells (49).

Other broadly expressed fibroblast-derived factors that regulate epithelial regeneration include growth factors, cytokines, and ECM molecules. Nrg1, an EGF family ligand, is upregulated in PDGFRα^+^ cells following irradiation and chemotherapy-induced injury and promotes intestinal cell proliferation and tissue repair through MAPK and AKT pathways (50). IL-33 produced by pericryptal fibroblasts protects against Salmonella infection by promoting epithelial cell differentiation (51). Angiopoietin-like protein 2 (Angptl2) is expressed in intestinal mesenchymal cells and regulates BMP expression to facilitate epithelial restoration following DSS- or irradiation-induced damage (52). Igf2bp1-mediated Ptgs2 expression by wound-associated fibroblasts is necessary for epithelial repair (53). Stromal-derived Ptgs2 and downstream PGE2 are activated by Tpl2 in response to innate stimuli to promote compensatory proliferation and improved intestinal healing upon TNBS- and DSS-mediated epithelial injury (54). Regulation of IGF1 signaling by stromal-specific miR-143/145 following DSS-induced injury promotes epithelial wound healing (55). ETS1-mediated ISLR secretion by stromal cells in the DSS- and TNBS-challenged murine intestine dampens Hippo signaling and enhances YAP in epithelial cells to facilitate regeneration and repair (56). Heat-Shock Factor 1 (HSF1) in colonic fibroblasts regulates ECM remodeling and thus crypt number and size during DSS colitis (57). The proteoglycan Asporin expressed by pericryptal fibroblasts promotes epithelial regeneration ex vivo and *in vivo* after chemotherapy-induced damage by inducing fetal-like state reversion in epithelial cells *via* activation of the Tgfβ signaling pathway (58). Finally, direct physical interaction, mediated through the generation of αSMA contractile stress fibers and deposition of collagen paths by fibroblasts, can orchestrate the organized and directed movement of epithelial cells and drive gap closure in an ex vivo model of intestinal wound healing (59).

#### 2.2.3 Fibroblasts regulate immune cell responses

Bulk, subset-specific, and single cell gene expression analyses have shown that upon damage or inflammation all fibroblast subsets express pro-inflammatory genes and could thus affect immune cell recruitment and function. *In vitro* studies also support the robust activation of inflammatory mediators, including cytokines, chemokines, and matrix remodeling enzymes, in intestinal fibroblasts in response to a variety of stimuli (7, 35, 36). Notably, these properties are in many studies attributed to intestinal (subepithelial) myofibroblasts, but this mainly reflects their *in vitro* morphology and expression of αSMA due to the culturing conditions, and it is thus impossible to ascribe them to specific subsets. IL-1β and TNF are the most well-established inducers of the pro-inflammatory activation of intestinal fibroblasts (20, 35, 54). IL-1β signaling specifically activates fibroblast-derived neutrophil-attracting factors and IL-1β co-localizes with FAP staining in ulceration sites of human patients (20). TNF also drives proinflammatory gene expression and fibroblast-restricted activation of TNF signaling is sufficient for the development of intestinal pathology in *TNF^ΔARE^* mice (60). In addition, Oncostatin M also induces chemokine production by stromal cells, leading to CD4^+^ T cell and granulocyte recruitment, which drive inflammation in a preclinical model of anti-TNF-resistant colitis (61). Besides cytokines, intestinal fibroblasts are also activated *via* TLR and NOD receptors, indicating their potential role as sentinel cells (35, 62). NFκB and MAPK signaling pathways are crucial downstream mediators of fibroblast activation. NFκB signaling, in particular, plays an important role in the activation of PDGFRα^hi^ fibroblasts during DSS colitis, as Col6a1-Cre-specific deletion of IKK2 led to reduced colitis, associated with decreased production of inflammatory mediators, reduced inflammatory cell infiltration and epithelial-specific STAT3 activation (63). However, deletion of IKK2 in Col1a2^+^ fibroblasts did not affect colitis development (64), suggesting that different signaling pathways could regulate the inflammatory activation of distinct subsets.

In addition to pro-inflammatory functions, intestinal fibroblasts can also regulate the resolution of inflammation through multiple paracrine mechanisms. For example, CCL2 secretion by colonic stromal cells in response to NOD2 activation by *C. rodentium* infection drives the recruitment of Ly6^hi^ inflammatory monocytes, which promote bacterial clearance (65). NLRP6 in colonic fibroblasts mediates tissue recovery through paracrine signaling that regulates epithelial cell proliferation and Th-17 immune responses (66). PD-L1 upregulation by fibroblasts in ulcerative colitis can suppress CD4^+^ T-cell activation, pathogenic IFN-γ secretion and Th1 responses (39, 67, 68). IL-36-mediated proliferation and cytokine/chemokine gene expression in colonic fibroblasts during the regeneration phase of acute colitis induces *in vitro* neutrophil migration to promote wound healing (69). Seprina3n, a serine protease inhibitor, secreted by stromal fibroblasts during the remission phase of DSS-induced inflammation, inhibits the function of elastase in recruiting neutrophils to the colon and as such facilitates the resolution of inflammation that could otherwise become pathogenic (43).

## 3 Discussion

In conclusion, recent studies, especially ones using single cell transcriptomics, have revealed distinct fibroblast subsets that regulate epithelial homeostasis along the crypt/villous axis and are similar between intestinal regions and across mammalian species. They also suggest that specific subsets can act as sources of more differentiated fibroblasts. Upon intestinal damage, fibroblasts are activated and support immune cell infiltration and function, while during repair they facilitate the resolution of inflammation and the tissue’s re-epithelization and morphogenesis through multiple mechanisms, although the subset and location-specificity of these functions is not clear. Further studies are expected to elucidate the plasticity of resident fibroblasts, their fates during inflammation and regeneration and their potential utility in the diagnosis and/or therapy of intestinal disorders.

## Author contributions

NC and CP wrote the manuscript and prepared the table and figure. VK critically revised the manuscript. All authors read and approved the submitted version.

## Funding

This work was supported by grants from the Worldwide Cancer Research (Grant No: 22-0126) and the Hellenic Foundation for Research & Innovation (HFRI) (Grant No: 3001) to VK.

## Conflict of interest

The authors declare that the research was conducted in the absence of any commercial or financial relationships that could be construed as a potential conflict of interest.

## Publisher’s note

All claims expressed in this article are solely those of the authors and do not necessarily represent those of their affiliated organizations, or those of the publisher, the editors and the reviewers. Any product that may be evaluated in this article, or claim that may be made by its manufacturer, is not guaranteed or endorsed by the publisher.
